# Frequency-tunable toughening in a polymer-metal-ceramic stack using an interfacial molecular nanolayer

**DOI:** 10.1038/s41467-018-07614-y

**Published:** 2018-12-07

**Authors:** Matthew Kwan, Muriel Braccini, Michael W. Lane, Ganpati Ramanath

**Affiliations:** 1 Materials Science and Engineering Department, Rensselaer Polytechnic Institute, Troy, NY 12180 USA; 2 SIMaP, Grenoble INP, CNRS, Univ. Grenoble Alpes, F-38000 Grenoble, France; 3 Chemistry Department, Emory and Henry College, Emory, VA 24327 USA

## Abstract

Interfacial toughening in composite materials is reasonably well understood for static loading, but little is known for cyclic loading. Here, we demonstrate that introducing an interfacial molecular nanolayer at the metal-ceramic interface of a layered polymer-metal-ceramic stack triples the fracture energy for ~75–300 Hz loading, yielding 40% higher values than the static-loading fracture energy. We show that this unexpected frequency-dependent toughening is underpinned by nanolayer-induced interface strengthening, which facilitates load transfer to, and plasticity in, the polymer layer. Above a threshold interfacial bond strength, the toughening magnitude and frequency range are primarily controlled by the frequency- and temperature-dependent rheological properties of the polymer. These results indicate the tunability of the toughening behavior through suitable choice of interfacial molecular layers and polymers. Our findings open up possibilities for realizing novel composites with inorganic-organic interfaces, e.g., arresting crack growth or stimulating controlled fracture triggered by loads with specific frequency characteristics.

## Abstract (web summary)

The toughening of layered composite materials during cyclic loading remains poorly understood. Here, the authors introduce an interfacial nanolayer to a polymer-metal-ceramic stack to triple the fracture energy during cyclic loading via nanolayer-induced interfacial bond strengthening and load transfer to the polymer layer.

## Introduction

Tailoring the chemistry of heterointerfaces is crucial to controlling the fracture toughness of a variety of composite materials, such as, those used in load-bearing structures^1^, nanoelectronics devices^2^, energy systems^3^, and biomedicine^4^. Interfacial fracture can occur at significantly lower stresses than the static-loading fracture stresses^5,6^ of the materials comprising the interface, and can be exacerbated by chemical attack (stress corrosion) and cyclic loading (fatigue), thereby adversely impacting reliability and performance. Although fatigue has been widely investigated and well understood in bulk materials^7,8^, much less is known about fatigue-induced interfacial fracture, especially in coatings and thin films. Recent works have examined the effects of chemical treatment^9,10^, patterning^11^, micrometer-thick adhesion layers^4,10^, crack-tip blunting^12^, and cyclic loading amplitude^13^ on interfacial fatigue, and described the results in terms of Paris law-based bulk-fatigue models^14,15^. But, loading-frequency-dependence of interfacial fatigue, and related phenomena, remain largely unexplored. Our prior work has shown that introducing an interfacial molecular nanolayer (MNL) in model polymer-metal-ceramic structures can yield multifold increases in fracture energy under static loading^16–19^, enhance thermal^20^ and electronic^21–25^ transport, inhibit diffusion^26–28^, and alter phase formation^29^. However, the effects of cyclic loading on the fracture behavior of such molecularly modified model systems are not known. Understanding frequency-dependent effects in molecularly modified structures, such as, accelerated damage, crack growth mitigation, and interfacial healing, is not only of fundamental importance but also should enable the design of smart composites^30–32^ comprised of soft-hard and/or organic-inorganic interfaces for emerging applications in electronics, energy, and biomedicine.

Here we report loading-frequency-dependent multifold fracture toughening upon inserting a strongly binding MNL at the metal-ceramic interface of a layered polymer-metal-ceramic stack. The interfacial MNL results in up to threefold higher fracture energy in the ~75–300 Hz range than the invariant value at other frequencies. We demonstrate that this remarkable behavior is underpinned by MNL-induced interface strengthening that enables load transfer to, and plasticity in, the distal polymer layer. Furthermore, the magnitude and frequency range of fatigue toughening correlates with the rheological properties of the polymer, varied by altering the temperature relative to the polymer glass transition. Our findings suggest that fatigue fracture energy is tunable by appropriate choices of MNL(s) and polymer(s), opening up a completely new set of possibilities to tailor composite materials. For example, the interface can be tailored to shift from a crack growth mode to crack arrest mode, or controllably fracture in response to stimuli with specific loading-frequency characteristics.

## Results

### Experimental work

**Fig. 1:**
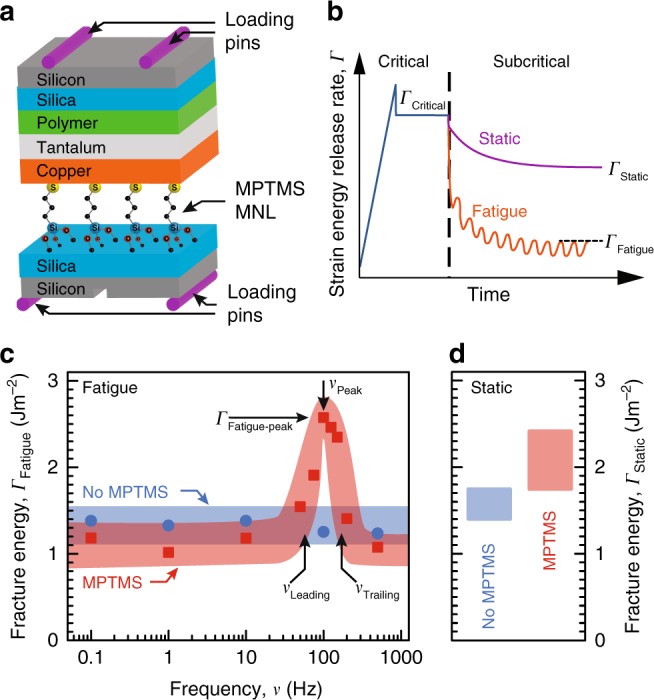
Fracture energy of epoxy-Cu-MPTMS-SiO_2_ structures under static and fatigue loading. **a** Schematic depicting the four-point bending test and **b** the strain energy release rate characteristics for load-shedding fatigue and static loading. **c** Fatigue fracture energy of polymer-metal-ceramic structures with Cu-MPTMS-SiO_2_ interfaces (red squares) and Cu-SiO_2_ interfaces (blue circles) determined at $p_{{\mathrm{H}}_{2}{\mathrm{O}}}$ = 0.6 kPa, shown together with **d** the corresponding static stress fracture energies. Each data point represents at least three tests. The width of the bands, drawn through the data points to guide the eye, connote the experimental uncertainty measured as standard deviation

We prepared polymer-metal-MNL-ceramic structures sandwiched between two silica-capped Si(001) wafers for four-point-bend mechanical tests^19^ (Fig. 1a). We self-assembled a mercapto-propyl-tri-methoxysilane (MPTMS) MNL on a silica-capped Si wafer surface. We then sputter-deposited a 40-nm-thick Cu layer with a 7 mTorr Ar plasma in a 5 × 10^−7^ Torr base pressure CVC tool. Without breaking vacuum, we also deposited a 100-nm-thick Ta layer to facilitate metal-polymer bonding ^17^. We glued the metal-MNL-silica structures to a dummy Si wafer with a System Three Resins^®^ T88 epoxy polymer to obtain beams comprised of layered Si-polymer-Ta-Cu-MPTMS-SiO_2_-Si structures for four-point-bend mechanical tests^19^ (Fig. 1a). We also created and tested beams without the MNL (see Methods).

Four-point bend tests were carried out under fatigue and static loading at temperatures ranging from 15 ≤ *T* ≤ 50 °C and at preset water partial pressures between 0.6 ≤  $p_{{\mathrm{H}}_{2}{\mathrm{O}}}$ ≤ 2.8 kPa. A Physik Instrumente P216-9S piezo actuator was used to produce load-cycling. Following crack initiation of the Cu-SiO_2_ interface^33^ at a critical strain energy release rate (*Γ*_Critical_), we conducted displacement-controlled subcritical (i.e., *Γ* < *Γ*_Critical_) load-shedding tests^18,34^. Example stress-time curves from a load-shedding fatigue test, and a static-loading test, are schematically depicted in Fig. 1b. For the fatigue tests, we chose a displacement-amplitude between 5 and 30 μm to obtain an initial stress slightly higher than that corresponding to the fatigue fracture energy *Γ*_Fatigue_ to be measured. We applied sinusoidal load oscillations in the 0.1 ≤ *ν* ≤ 1000 Hz range, with a maximum-to-minimum load ratio ~ 10. We extracted the *Γ*_Fatigue_ from strain energy release rate-crack velocity (*Γ*-*u*_crack_) plots by recognizing that *Γ* = *Γ*_Fatigue_ at *u*_crack_ = 0. We also determined the stress corrosion fracture energy *Γ*_Static_ from static load tests^19^ to explicitly separate the effects of chemical attack and load-cycling. The fracture surfaces were examined by X-ray photoelectron spectroscopy (XPS) and polarized-light microscopy.

### Loading-frequency-dependent toughening

Our results show that introducing a MPTMS nanolayer at the Cu-SiO_2_ interface of our polymer-metal-silica stacks significantly influences the fatigue fracture behavior. Stacks without the MPTMS nanolayer exhibit a *Γ*_Fatigue_ ~ *Γ*_Static_ = 1.6 Jm^−2^ at 25 °C and $p_{{\mathrm{H}}_{2}{\mathrm{O}}}$ = 0.6 kPa (Fig. 1c), irrespective of the loading frequency. Inserting an MPTMS nanolayer at the Cu-SiO_2_ interface produces a 30% higher *Γ*_Static_ = 2.1 Jm^−2^, as expected^35^ (Fig. 1d). But, MPTMS functionalization decreases the fatigue fracture energy to *Γ*_Fatigue_ ~ 1.1 Jm^−2^ (i.e., *Γ*_Fatigue_ < *Γ*_Static_) at all frequencies, except *ν* ~ 75–125 Hz, where we observe *Γ*_Fatigue_ > *Γ*_Static_ and a maximum of *Γ*_Fatigue-peak_ ~ 2.6 Jm^−2^. In particular, *Γ*_Fatigue_ increases above a threshold frequency *ν*_Leading_, which we refer to as the leading edge (Fig. 1c). For *ν* > *ν*_Leading_, *Γ*_Fatigue_ goes through a maximum at *ν*_max_, and decreases at higher *ν*. Fatigue toughening is not detectable for *ν* > *ν*_Trailing_, where *ν*_Trailing_ corresponds to the trailing edge of the *Γ*_Fatigue_ peak, i.e., where *Γ*_Fatigue_ < *Γ*_Static_.

Our experiments revealing *Γ*_Fatigue_ < *Γ*_Static_ at very low and very high load-cycling frequencies is not unexpected because subcritical cyclic loading is known to hasten interfacial fracture^11^. However, the observed loading-frequency-dependent toughening at intermediate frequencies (i.e., *ν*_Leading_ ≤ *ν* ≤ *ν*_Trailing_) indicated by the *Γ*_Fatigue_ peak in the MPTMS-modified structures, is unusual. This result indicates that molecular functionalization of the weakest interface can actually increase the fracture energy at certain loading frequencies, to values higher than the static-loading fracture energy.

**Fig. 2:**
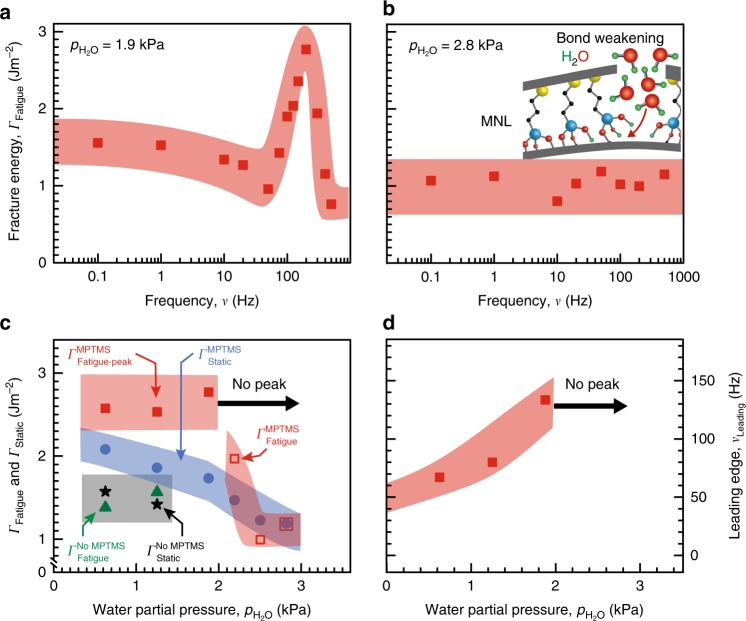
Effect of moisture-dependent interfacial bond strength on fatigue toughening. Fatigue fracture energy *Γ*_Fatigue_ of polymer-metal-ceramic structures with Cu-MPTMS-SiO_2_ interfaces shown for water partial pressures **a** $p_{{\mathrm{H}}_{2}{\mathrm{O}}}$ = 1.9 kPa and **b** $p_{{\mathrm{H}}_{2}{\mathrm{O}}}$ = 2.8 kPa. **c** *Γ*_Fatigue_ peak value, i.e., *Γ*_Fatigue-peak_ (red squares) and average *Γ*_Static_ (blue circles) plotted versus $p_{{\mathrm{H}}_{2}{\mathrm{O}}}$ for stacks with Cu-MPTMS-SiO_2_ interfaces. Average *Γ*_Fatigue_ (green triangles) and *Γ*_Static_ (black stars) for stacks without MPTMS are also shown. There is no observable *Γ*_Fatigue_ peak for $p_{{\mathrm{H}}_{2}{\mathrm{O}}}$ > 2.2 kPa (open red squares). **d** Leading edge frequency *ν*_Leading_ of the *Γ*_Fatigue_ peak for Cu-MPTMS-SiO_2_ interfaces. No *Γ*_Fatigue_ peak is observed for $p_{{\mathrm{H}}_{2}{\mathrm{O}}}$ ≥ 2.2 kPa. Each data point represents at least three tests. The width of the bands, drawn through the data points to guide the eye, connote the experimental uncertainty measured as standard deviation

In order to understand the MPTMS-induced fatigue toughening, we measured the fracture energy as a function of the water-sensitive siloxane bond strength^36^ at the Cu-MPTMS-SiO_2_ interface by adjusting the water partial pressure $p_{{\mathrm{H}}_{2}{\mathrm{O}}}$. For low $p_{{\mathrm{H}}_{2}{\mathrm{O}}}$ < 2.2 kPa we obtain a *Γ*_Fatigue_ peak at *ν* ~ 75–300 Hz in structures with Cu-MPTMS-SiO_2_ interfaces (Fig. 2a). At higher $p_{{\mathrm{H}}_{2}{\mathrm{O}}}$ ≥ 2.2 kPa *Γ*_Fatigue_ is essentially invariant in the 0.1 ≤ *ν* ≤ 500 Hz range, with no observable fatigue toughening (Fig. 2b), reflecting a behavior similar to that in structures without MPTMS. Since moisture weakens siloxane bonds, our results indicate a minimum interface bonding strength, attainable below a threshold $p_{{\mathrm{H}}_{2}{\mathrm{O}}}$, is a prerequisite for fatigue toughening. This is reminiscent of static toughening at Cu-MPTMS-SiO_2_ interfaces below a threshold $p_{{\mathrm{H}}_{2}{\mathrm{O}}}$ that provides adequate siloxane bonding strength to enable metal plasticity^18,19^.

Unlike a monotonic increase in the magnitude of static toughening with desiccation, fatigue toughening increases with desiccation in the 1.9 kPa ≤ $p_{{\mathrm{H}}_{2}{\mathrm{O}}}$ ≤ 2.5 kPa range, but saturates at *Γ*_Fatigue_ = *Γ*_Fatigue-peak_ for $p_{{\mathrm{H}}_{2}{\mathrm{O}}}$ < 1.9 kPa and is insensitive to further desiccation (Fig. 2c). We note that *Γ*_Fatigue_ > *Γ*_Static_ at $p_{{\mathrm{H}}_{2}{\mathrm{O}}}$ = 2.2 kPa, despite the absence of a *Γ*_Fatigue_ peak. These observations suggest that the *Γ*_Fatigue-peak_ magnitude is limited by a mechanism other than desiccation-induced interfacial siloxane bond strengthening. Fatigue toughening occurs only below a threshold $p_{{\mathrm{H}}_{2}{\mathrm{O}}}$ and only for *ν* ≥ *ν*_Leading_, indicating that *ν*_Leading_ corresponds to the threshold $p_{{\mathrm{H}}_{2}{\mathrm{O}}}$ at which the minimum required siloxane bond strength is attained at the crack tip. Toughening is precluded for *ν* < *ν*_Leading_ because of facile water-induced siloxane bond-breaking due to a higher $p_{{\mathrm{H}}_{2}{\mathrm{O}}}$ at the crack tip than the threshold $p_{{\mathrm{H}}_{2}{\mathrm{O}}}$. Conversely, *ν* > *ν*_Leading_ corresponds to a lower $p_{{\mathrm{H}}_{2}{\mathrm{O}}}$ at the crack tip than the threshold $p_{{\mathrm{H}}_{2}{\mathrm{O}}}$. Thus, increasing frequency for *ν* > *ν*_Leading_ (or equivalently, downshifting *ν*_Leading_) is tantamount to crack-tip desiccation. This is indeed corroborated by our results showing desiccation-induced downshifting of *ν*_Leading_ below $p_{{\mathrm{H}}_{2}{\mathrm{O}}}$ ≤ 1.9 kPa (Fig. 2d).

The correlations between increasing *ν* and desiccation suggest that the water molecules are increasingly hindered from reaching the crack tip for *ν* > *ν*_Leading_, similar to that reported for high loading rates^37–39^. The build-up of elastic energy in the unbroken interfacial bonds increases the interfacial work of adhesion *γ*_a_ and becomes available for activating plastic energy dissipation *γ*_p_ in the adjacent layers. Thus, siloxane bond strength is the limiting determinant of the toughening magnitude with increasing frequency in the *ν*_Leading_ ≤ *ν* ≤ *ν*_peak_ range and desiccation in the 1.9 kPa ≤ $p_{{\mathrm{H}}_{2}{\mathrm{O}}}$ ≤ 2.8 kPa range. Decreasing *Γ*_Fatigue_ for *ν* > *ν*_peak_ and the invariance of *Γ*_Fatigue-peak_ magnitude for $p_{{\mathrm{H}}_{2}{\mathrm{O}}}$ < 1.9 kPa are contrary to desiccation-induced bond strengthening, confirming that *Γ*_Fatigue_ at high *ν* and low $p_{{\mathrm{H}}_{2}{\mathrm{O}}}$ is not limited by the interfacial strength.

### Interfacial fracture and plastic energy dissipation

**Fig. 3:**
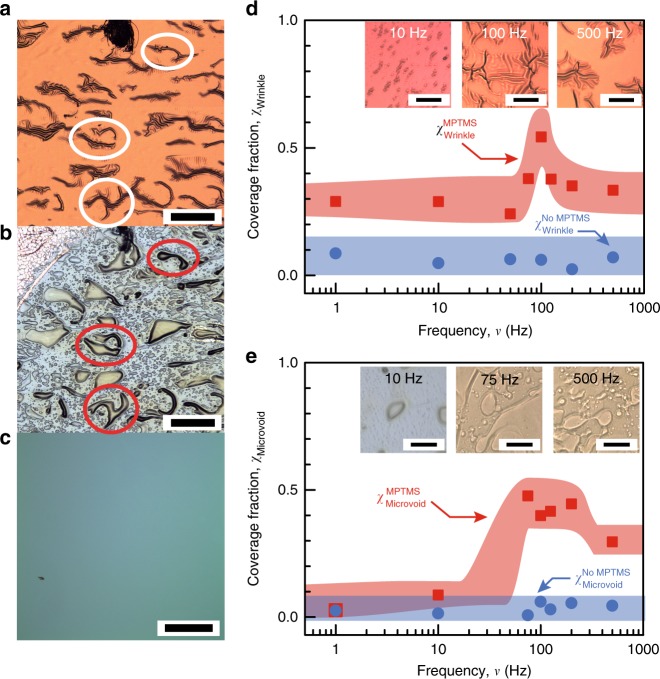
Fracture surface analyses of epoxy-Cu-MPTMS-SiO_2_ structures under fatigue loading. **a** Representative optical micrographs from an as-obtained Cu fracture surface (scale bar = 300 μm), **b** the same with the metal film etched off to expose the polymer (scale bar = 300 μm), and **c** the SiO_2_ fracture surface (scale bar = 100 μm). The sizes and shapes of the metal wrinkles and the polymer microvoids are correlated (circles regions). Average **d** metal wrinkle coverage *χ*_Wrinkle_ (scale bar = 100 μm) and **e** microvoid coverage *χ*_Microvoid_ (scale bar = 200 μm) from fracture surfaces of structures with (red squares), and without (blue circles) MPTMS. Representative optical micrographs inset in **d** and **e** capture metal wrinkling and polymer microvoiding at select frequencies. The data in this figure were from experiments at $p_{{\mathrm{H}}_{2}{\mathrm{O}}}$ = 1.3 kPa. The width of the bands drawn through the data points connote the experimental uncertainties measured as standard deviation

Fracture surface analyses suggests that MPTMS-functionalization leads to fatigue toughening by facilitating plasticity in the metal-polymer bilayer. Core-level XPS spectra from fracture surfaces confirm that fatigue fracture occurs via siloxane bond-breaking at the MPTMS-silica interface (Supplementary Fig. 1), as reported for static loading^17,18^. Polarized-light micrographs of Cu fracture surfaces exhibit 5- to 20-μm-scale wrinkles (Fig. 3a). Etching off the metal film reveals microvoids in the polymer that are similar in shape and size of the metal wrinkles at the same location (Fig. 3b), suggesting that polymer voiding and metal wrinkling are correlated. In contrast, the silica fracture surfaces were featureless (Fig. 3c), consistent with our XPS analysis. Load-cycling results increases the average microvoid area *ζ*_Microvoid_ multifold, e.g., from 175 to 980 μm^2^ (Supplementary Fig. 2). Radially oriented polarization fringes around the microvoids (Supplementary Fig. 3) indicate microvoid growth by shear banding involving the back-and-forth motion of polymer chains^9^. Fracture surfaces obtained by static loading show neither metal wrinkling nor polymer microvoid growth, confirming that these features arise from load-cycling-induced plasticity.

Both metal wrinkling and polymer microvoid coverages (*χ*_Wrinkle_ and *χ*_Microvoid_) are dependent on the loading frequency. We observe *χ*_Wrinkle_ peaks between ~75 and 125 Hz (Fig. 3d) with a 60% higher coverage than other frequencies, while *χ*_Microvoid_ increases eightfold and peaks over a wider frequency range of ~75–200 Hz, and saturates at a slightly lower value above ~200 Hz (Fig. 3e). The frequency regimes of the coverage peaks correlate well with the 75–300 Hz regime of the *Γ*_Fatigue_ peak. In contrast, fatigue fracture surfaces of stacks without MPTMS exhibit ninefold lower *χ*_Microvoid_ and fourfold lower *χ*_Wrinkle_, both of which are invariant with frequency. These results confirm that MPTMS-induced interfacial strengthening is key to activating loading-frequency-dependent plasticity in the polymer-metal bilayer.

The greater overlap in the frequency regime of the *χ*_Microvoid_ peak and the *Γ*_Fatigue_ peak suggests a greater contribution of polymer plasticity to the observed fatigue toughening. Cu film strain, estimated from *χ*_Wrinkle_ and wrinkle amplitude^40^ measurements (Supplementary Fig. 3), indicates a metal plastic energy *U*_m_ ~ 0.02 ± 0.01 Jm^−2^, which accounts for <1% of the fracture energy at *Γ*_Fatigue-peak_ ~ 2.5 Jm^−2^ (Supplementary Methods). Such low metal plasticity is attributable to the high-yield stress Cu film that results in a highly confined plastic zone near the crack tip vicinity. This view is consistent with our estimates^19^ of <~2% plastic strain energy and a <1 nm plastic zone for a 40-nm-thick Cu film used in our experiments here. Thus, fatigue toughening observed in our experiments is underpinned primarily by polymer plasticity, while the metal film essentially serves as an elastic stress-transfer layer. We note that this mechanism is unlike MNL-induced metal-ceramic interface toughening due to metal plasticity under static loading^19^.

**Fig. 4:**
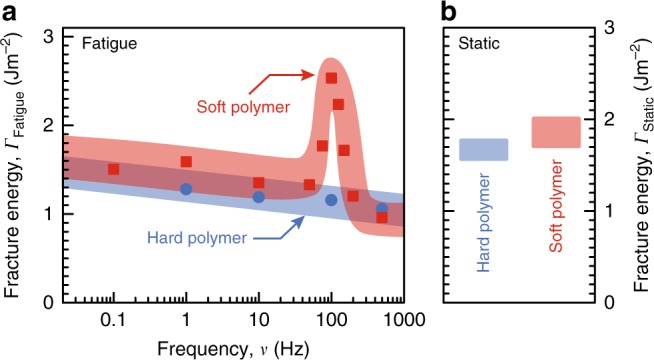
Effect of polymer hardness on fatigue toughening. **a** Fatigue fracture energy *Γ*_Fatigue_ and **b** *Γ*_Static_ of polymer-metal-MPTMS-SiO_2_ stacks with a soft System Three Resins^®^ T88 polymer (red squares), and a harder EPO-TEK 375 polymer (blue circles) at $p_{{\mathrm{H}}_{2}{\mathrm{O}}}$ = 1.3 kPa. Each data point represents at least three tests. The width of the bands, drawn through the data points to guide the eye, connote the experimental uncertainties measured as standard deviation

We propose that microvoid growth in the polymer leads to metal-polymer interface delamination at the voids. The consequent release of constraint leads to metal film wrinkling due to compressive stresses^41^. This hypothesis is supported by the disappearance of fatigue toughening and metal wrinkling when polymer plasticity is suppressed by replacing the T88 epoxy with the harder EPO-TEK 375 epoxy in our structures (Fig. 4 and Supplementary Fig. 3). The *Γ*_Fatigue_ peak frequency range is also consistent with the toughening of T88 epoxy composites^42–45^ at strain rates of ~0.02–0.1 s^−1^ (Supplementary Methods). If voiding were to initiate at the metal-polymer interface, we would expect significant metal wrinkling under static loading, and a fracture path change from the MNL-SiO_2_ interface, neither of which we observe. These results indicate that MPTMS-induced polymer plasticity is the primary fatigue toughening mechanism.

### Tuning the toughening magnitude and frequency range

In order to understand fatigue toughening magnitude and frequency characteristics in terms of polymer rheology, we examined the *Γ*_Fatigue_ peak at different temperatures below the polymer glass transition temperature *T*_g_. Since *T*_g_ for our T88 epoxy is frequency-dependent (e.g., 52 ≤ *T*_g_ ≤ 75 °C between 0.01 ≤ *ν* ≤ 1000 Hz (Supplementary Fig. 4), we analyzed temperature-dependent toughening in terms of Δ*T* = *T*_g_ − *T* for data acquired at a fixed $p_{{\mathrm{H}}_{2}{\mathrm{O}}}$ = 1.3 kPa. Separate measurements of polymer film stress on SiO_2_ indicate that water-induced polymer swelling is insignificant, e.g., <~3% of the compliance change seen during crack growth (Supplementary Fig. 5).

**Fig. 5:**
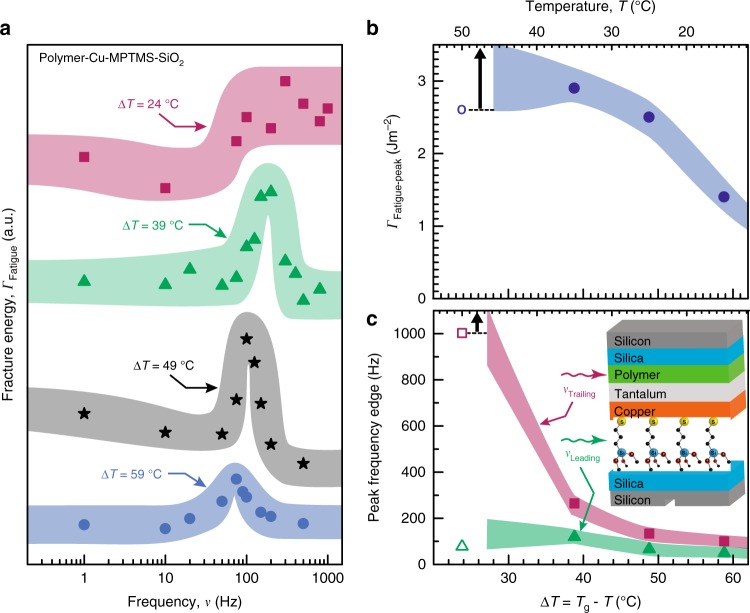
Temperature-dependence of the fatigue toughening magnitude and frequency characteristics. **a** Fatigue fracture energy *Γ*_Fatigue_ of polymer-metal-MPTMS-silica interfaces for 24 °C ≤ Δ*T* ≤ 59 °C, at $p_{{\mathrm{H}}_{2}{\mathrm{O}}}$ = 1.3 kPa. **b** The *Γ*_Fatigue_ trailing edge frequency *ν*_Trailing_ (magenta squares), leading edge frequency *ν*_Leading_ (green triangles), and **c** *Γ*_Fatigue_ peak height *Γ*_Fatigue-peak_ (blue circles), plotted as a function of Δ*T*. The *Γ*_Fatigue-peak_ and *ν*_Trailing_ values shown for Δ*T* = 24 °C represent the lower bounds (open symbols) because the *ν*_Trailing_ edge at this temperature extends beyond our instrument frequency range. Each data point in **b** and **c** is extracted from the corresponding curve in **a**. The width of the bands, drawn through the data points to guide the eye, connote the experimental uncertainties measured as standard deviation

We find that lower Δ*T* (i.e., higher *T*, closer to *T*_g_) correlates with a higher *Γ*_Fatigue-peak_, and a larger peak width (Δ*ν* = *ν*_Trailing_ − *ν*_Leading_), indicating that both the fatigue toughening magnitude and frequency range are sensitive to polymer plasticity (Fig. 5a). For instance, a 20 °C increase in temperature from 16 to 36 °C (i.e., decreasing Δ*T* from 59 to 39 °C) doubles the *Γ*_Fatigue_ peak magnitude to *Γ*_Fatigue-peak_ = 2.9 Jm^−2^ (Fig. 5b), which is 40% higher than the highest static-loading fracture energy *Γ*_Static_ measured at $p_{{\mathrm{H}}_{2}{\mathrm{O}}}$ = 0.6 kPa (Fig. 2c). This *Γ*_Fatigue-peak_ doubling correlates with the doubling of the loss-to-storage moduli ratio tan *δ* of the epoxy for the same temperature increase (Supplementary Fig. 4), suggesting that polymer rheology determines the *Γ*_Fatigue-peak_ magnitude. Since tan *δ* increases with temperature for *T* < *T*_g_, but decreases with frequency (Supplementary Fig. 4b-c), polymer plasticity is facilitated at higher temperatures but deterred at higher frequencies. Increasing *Γ*_Fatigue-peak_ magnitude with temperature and decreasing *Γ*_Fatigue_ for *ν* > *ν*_Peak_ mirror the tan *δ* behavior, confirming that polymer plasticity is the predominant contributor to the observed fatigue toughening. Thus, decreasing polymer plasticity at *ν* > *ν*_Peak_ counteracts increases in interfacial strength at *ν* > *ν*_Leading_, leading to a fatigue toughening maximum.

Decreasing Δ*T* (i.e., increasing *T*, closer to *T*_g_) also extends fatigue toughening to higher frequencies. Both *ν*_Leading_ and *ν*_Trailing_ shift to higher frequencies, but the *ν*_Trailing_ shift is ~120% greater (Fig. 5c). The temperature-induced *ν*_Trailing_ shifts can be understood by recognizing that plasticity is arrested above *ν*_Trailing_ due to the diminished responsiveness of polymer chains to high-frequency load-cycling, which is consistent with decreasing tan *δ* with increasing frequency. Higher chain mobility at temperatures closer to *T*_g_ (i.e., high *T* and low Δ*T*) enables greater polymer plasticity, which is manifest as a higher *Γ*_Fatigue-peak_ magnitude and shifting of the plasticity arrest point *ν*_Trailing_ to higher frequencies as Δ*T* decreases. Since *ν*_Leading_ corresponds to a threshold strength of the water-sensitive siloxane bonds, and $p_{{\mathrm{H}}_{2}{\mathrm{O}}}$ is held constant here, the up-shifts in *ν*_Leading_ with decreasing Δ*T* indicates that increasing the temperature enhances the transport of water molecules to the crack tip.

## Discussion

Based upon our results, the salient mechanistic aspects of the frequency-dependent toughening observed in polymer-metal-ceramic structures with an MNL-functionalized metal-ceramic interface can be understood as follows. Interfacial fracture energy has two main contributors: the metal-ceramic interface work of adhesion *γ*_a_, and plastic energy dissipation *γ*_p_ in the adjacent layers. Introducing the MPTMS MNL increases *γ*_a_ through siloxane bond formation at the MNL-SiO_2_ interface. Water attack of siloxane bonds lowers *γ*_a_. However, increasing load-cycling frequency curtails water transport to the crack tip, leading to an effective increase in *γ*_a_. Thus, increasing frequency is tantamount to desiccation, which strengthens siloxane bonds. The consequent build-up in interfacial elastic energy becomes available for activating plasticity (i.e., *γ*_p_ ≠ 0) in the adjacent layers. The minimum *γ*_a_ necessary for elastic energy build-up and plastic energy dissipation for detectable fatigue toughening is achieved at *ν*_Leading_, and increases for *ν* > *ν*_Leading_.

In our experiments, plasticity *γ*_p_ occurs mainly in the polymer by microvoid growth via shear banding. The high-yield stress metal film serves as an elastic load transfer layer, and wrinkles due to compressive stresses generated through release of constraint when microvoids reach the polymer-metal interface. Polymer plasticity itself, however, decreases with increasing frequency due to the increasing inability of polymer chain motion to keep up with load-cycling. Increasing *γ*_a_ and decreasing *γ*_p_ with frequency results in a fatigue toughening maximum, i.e., a *Γ*_Fatigue_ peak, at intermediate frequencies. The increase in *Γ*_Fatigue_ with frequency for *ν*_leading_ ≤ *ν* ≤ *ν*_Peak_ indicates that fatigue toughening is limited by *γ*_a_. At *ν* ≥ *ν*_Peak_, the decreasing contribution of *γ*_p_ due to inhibited chain motion begins to offset the frequency-induced increases in the elastic energy due to interfacial bond strengthening. Decreasing fatigue toughening for *ν* > *ν*_peak_ and saturation for $p_{{\mathrm{H}}_{2}{\mathrm{O}}}$ < 1.9 kPa are contrary to interfacial siloxane bond strengthening expected at high *ν* and low $p_{{\mathrm{H}}_{2}{\mathrm{O}}}$, confirming that fatigue toughening is limited by polymer plasticity at high frequencies.

Although bond strengthening *γ*_a_ through the use of the MNL is a necessary condition for fatigue toughening, polymer plasticity *γ*_p_ is the predominant contributor. This is clearly seen from the suppression of fatigue toughening in MNL-modified structures with a hard epoxy. Furthermore, the maximum toughening magnitude *Γ*_Fatigue-peak_ and high-frequency limit of toughening *ν*_Trailing_, are determined by the rheological properties of the polymer. For example, both *Γ*_Fatigue-peak_ and *ν*_Trailing_ increase with temperature (as *T* approaches *T*_g_) due to facile chain motion, which is counteracted to some extent by the frequency-induced increase in *T*_g_ at higher frequencies. Therefore, choosing a polymer with the appropriate rheological properties is crucial for tailoring the toughening magnitude as well as the high-frequency limit.

**Fig. 6:**
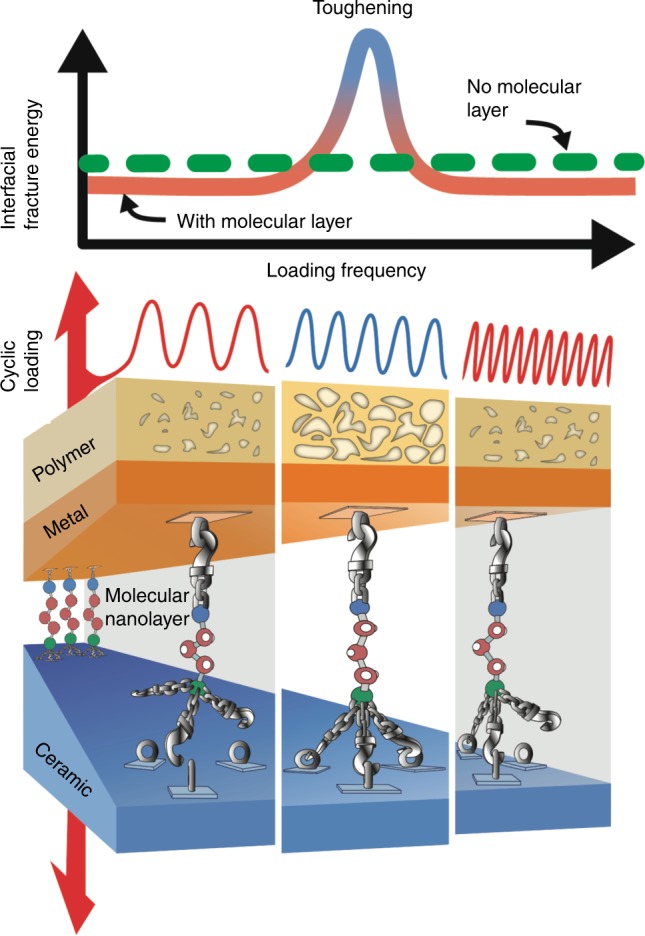
Frequency-dependent toughening enabled by interfacial strengthening and polymer rheology. Schematic sketch of loading-frequency-dependent interfacial fracture energy increase in an epoxy-Cu-MPTMS-SiO_2_ stack caused by plasticity in the polymer layer through voiding activated by MNL-induced interfacial strengthening. At low frequencies, water-induced siloxane bond-breaking at the MPTMS MNL-silica interface limits the interface fracture energy, and no polymer voiding is observed. At intermediate frequencies, the increased interface strength due to diminishing water attack at the crack tip facilitates load transfer to, and plasticity in, the polymer, yielding fatigue fracture energies exceeding the static-loading fracture energy; polymer voiding is observed. Arrested polymer plasticity (no voiding observed) due to curtailed chain mobility at very high loading frequencies leads to a low interfacial fracture energy despite high interfacial strength

Based upon the above, the overall mechanism of fatigue toughening of the epoxy-Cu-MPTMS-SiO_2_ polymer-metal-MNL-ceramic stack enabled by the MPTMS MNL can be understood in terms of Fig. 6. At low loading frequencies, water-induced siloxane bond-breaking at the MNL-ceramic interface limits the interface fracture energy. At intermediate loading frequencies, the increased interface strength caused by the diminishing effect of water attack at the MNL-SiO_2_ interface facilitates polymer plasticity resulting in fracture energies exceeding the static-loading fracture energy. Arrested polymer plasticity due to curtailed chain mobility at very high loading frequencies leads to the disappearance of polymer plasticity, and hence, fatigue toughening. Although the metal serves as an elastic layer in our experiments, using metals of different yield stresses, moduli, and/or thicknesses, may alter the contributions of metal and polymer plasticity, which could amplify, suppress, and/or modify the fatigue toughening frequency range.

Our findings are relevant to the design, monitoring, and controlling the stability of smart composites with tunable frequency-dependent toughening and/or weakening behaviors. For example, low-frequency fatigue toughening commencement *ν*_Leading_ in polymer-metal-MNL-ceramic structures can be controlled by suitable choice of MNLs with termini that enable strong interfacial bonding that supports sufficient interfacial elastic energy build-up for activating plasticity in the adjacent layers. Choosing polymers with appropriate viscoelastic properties should allow the tuning of the maximum toughening magnitude *Γ*_Fatigue-peak_ and high-frequency toughening limit *ν*_Trailing_. The use of multiple polymers with different rheological properties could result in novel *Γ*_Fatigue_-*ν* characteristics with multiple peaks, plateaus, valleys, and combinations thereof. Such frequency-dependent phenomena could pave the way for realizing novel composites that respond to select loading magnitude/frequency stimuli by either controllably degrading^46,47^, or self-healing^48^ through polymer plasticity and crosslinking of healing agents released during microvoid growth in the polymers.

In summary, we have shown that functionalizing polymer-metal-ceramic structures with a MNL at the metal-ceramic interface can lead to multifold increases in fracture energy at certain loading frequencies, yielding values higher than that obtained during static loading. Nanolayer-induced interfacial strengthening allows load transfer to, and plasticity in, the polymer layer. While a threshold interfacial strength determines the minimum loading frequency for toughening, the magnitude and loading-frequency range of toughening are primarily dependent on polymer rheology. These facets of heterointerfacial mechanics could be harnessed to design, monitor, and control the stability of composite materials for diverse applications including energy and electronics devices^46^, biomedicine^47^, and smart-degrading and self-healing systems^48^.

## Electronic supplementary material

Supplementary Information

## Data Availability

The data for the figures that support the findings of this study are available in figshare data repository with the identifier(s) 10.6084/m9.figshare.7154930 (fracture energy data), 10.6084/m9.figshare.7159529 (microscopy data), 10.6084/m9.figshare.7159541 (XPS data), and 10.6084/m9.figshare.7159553 (DMA data).
