# Supplementary material for: Frequency-tunable toughening in a polymer-metal-ceramic stack using an interfacial molecular nanolayer
Source: Nat Commun. 2018 Dec 7;9:5249. doi: 10.1038/s41467-018-07614-y (PMC6286376; doi:10.1038/s41467-018-07614-y)
Supplement: Supplementary file 1 — Supplementary Information [file 41467_2018_7614_MOESM1_ESM.pdf]

Frequency-tunable toughening in a polymer-metal-ceramic stack using an interfacial molecular nanolayer

Kwan et. al.

Supplementary Information

Supplementary Methods

Preparation of polymer-metal-ceramic structures

We prepared polymer-metal-MNL-ceramic structures sandwiched between two silica-capped Si(001) wafers for four point-bend mechanical tests[1] (Figure 1). We coated a mercapto-propyl-tri-methoxysilane (MPTMS) MNL via self-assembly onto silica surfaces by dipping the wafers into a 5 mM MPTMS solution in toluene. A 40±5 nm-thick Cu layer and a 100±5 nm-thick Ta overlayer were successively sputter-deposited onto the MNL prior to bonding the metal-MNL-ceramic structures to a dummy Si wafer with a ~18±10-μm-thick layer of a T88 epoxy from System Three Resins®. The Ta layer was used because the epoxy does not adhere well to Cu[2]. Thus, the metal is actually a Cu-Ta bilayer in the layered Si-T88-Ta-Cu-MPTMS-SiO_2-Si structures. We also created and tested reference beams without the MNL at the Cu-SiO_2 interface. The Si substrate was scribed with a 100 μm notch to initiate and propagate a crack to cause interfacial delamination during four-point bend testing.

In order obtain statistically significant trends amidst the stochastic nature of fracture, and large batch-to-batch variations in polymer film thickness, we conducted tests at different conditions (e.g., ν, p_{H_2O}) on samples taken from the same batch, and repeated such tests on at least two more batches. We also conducted tests at different un-cracked portions of the same sample under different conditions to benchmark variations within each batch. Our sample size and four-point bend instrument allow such retesting by adjusting the loading span, and the inner and outer load distances of the pins. The uncertainties indicated by bands in all the graphs denote data-spread due to batch-to-batch variations, which are larger than variations within the same-batch, and are uncorrelated with other test parameters.

The fatigue fracture energy Γ_{Fatigue} was determined from sub-critical (i.e., $\Gamma<\Gamma_{\text{Critical}}$) constant-displacement load-relaxation tests[3,4]. These load-shedding fatigue tests were carried out until the crack velocity u_{crack} (measured from the change in compliance of the beam during four-point-bend testing[4])

decayed to $\underline{u}_{\text{crack}} = 0$, at which point $\Gamma = \Gamma_{\text{Fatigue}}$. We also determined the stress corrosion fracture energy Γ_{Static} from similar load-shedding tests with static loads[1].

Fracture surface analyses

Analyses of Cu-MPTMS-SiO_2 fracture surfaces with a PHI 5400 X-ray photoelectron spectroscopy instrument reveal that both static- and cyclic-loading-induced fractures occur by siloxane bond fissure at the MPTMS-silica interface. In particular, the Cu fracture surface shows 101.9 eV Si 2p silyl-alkyl Si 2p signature[5] and a 162 eV S 2p Cu-S bond peak, indicative of MPTMS molecules tethered to Cu via Cu-S bonds (Supplementary Figure 1). Neither the silyl-alkyl moiety signature nor the S 2p peaks from MPTMS were detectable on the SiO_2 fracture surfaces, indicating that fracture occurs via Si-O-Si bond breaking at the MPTMS-SiO_2 interface.

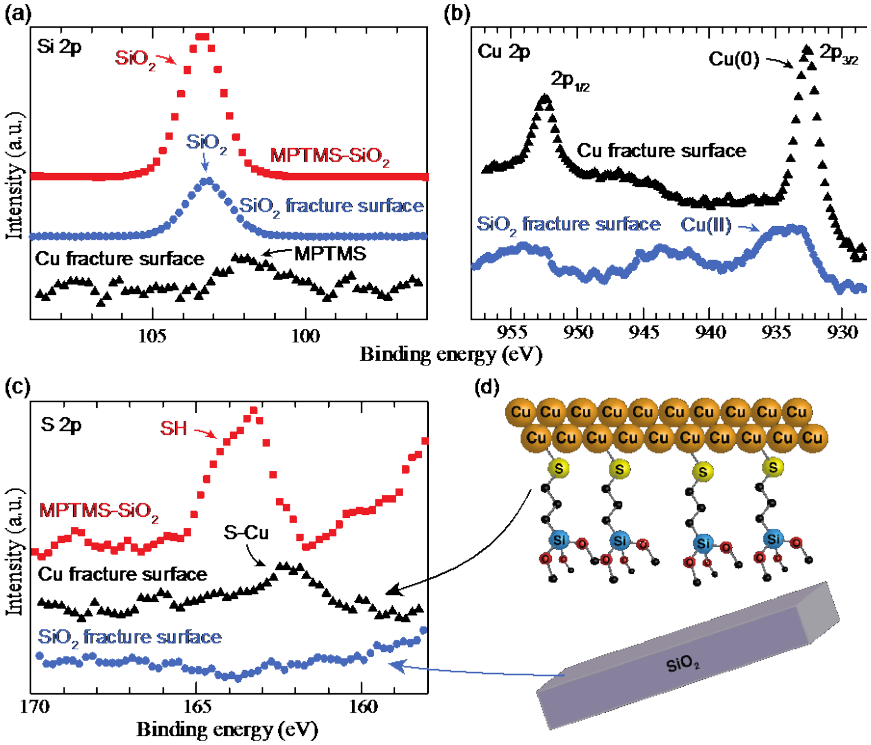

Supplementary Figure 1. X-ray photoelectron spectra in the vicinity of the (a) Si 2p, (b) Cu 2p, and (c) S 2p core level bands from the fracture surfaces of Cu-MPTMS-SiO_2 structures shown schematically in (d).

The metal and silica fracture surfaces were examined by polarized light microscopy. The presence of 15-25 μm wavelength metal wrinkles suggest plasticity due to compressive stresses[6]. Etching off the metal film with 10 μL drops of a 1:1 solution of 49% HF and 70% HNO_3 revealed microvoids in the underlying polymer. The microvoid size in the bulk polymer and polymer spread between glass slides served as a baseline for tracking microvoid growth during mechanical tests (Supplementary Figure 2). The The population of microvoids with areas $\zeta_{\text{Microvoid}} \geq 200\ \mu m^2$ is drastically diminished in fatigue-fractured polymer-metal-SiO_2 interfaces without MPTMS, the pre-test bulk polymer and spread polymer films. In contrast, fatigue-fractured polymer-metal-MPTMS-SiO_2 interfaces exhibit a near-even spread of $\zeta_{\text{Microvoid}}$ in the 0-2000 μm^2 range due to microvoid growth.

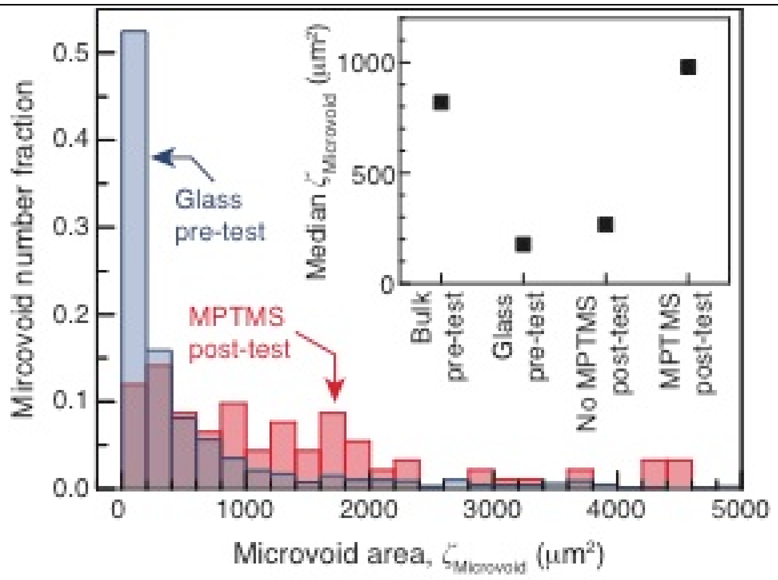

Supplementary Figure 2. Histograms of polymer microvoid area $\zeta_{\text{Microvoid}}$ from metal fracture surfaces of polymer-Cu-MPTMS-SiO_2 structures (MPTMS post-test) and pre-tested polymer spread between glass slides (Glass pre-test). Inset compares the median $\zeta_{\text{Microvoid}}$ for these, along with those for polymer-Cu-SiO_2 (No MPTMS post-test) and pre-tested bulk polymer (Bulk pre-test) samples.

Cross-polarized imaging with filters at 90° angles revealed radially-oriented shear bands around the microvoids (Supplementary Figure 3). We measured the coverages of the voids and wrinkles by tracing their perimeters in the optical micrographs and by analyzing the sizes and numbers of the resultant polygons using the imageJ software program. Neither metal wrinkling nor microvoid growth was observable from either fracture surfaces of structures without MPTMS, or under any of the following three conditions: the use of a harder polymer epoxy, static loading, and cyclic-loading on a pre-cracked interface.

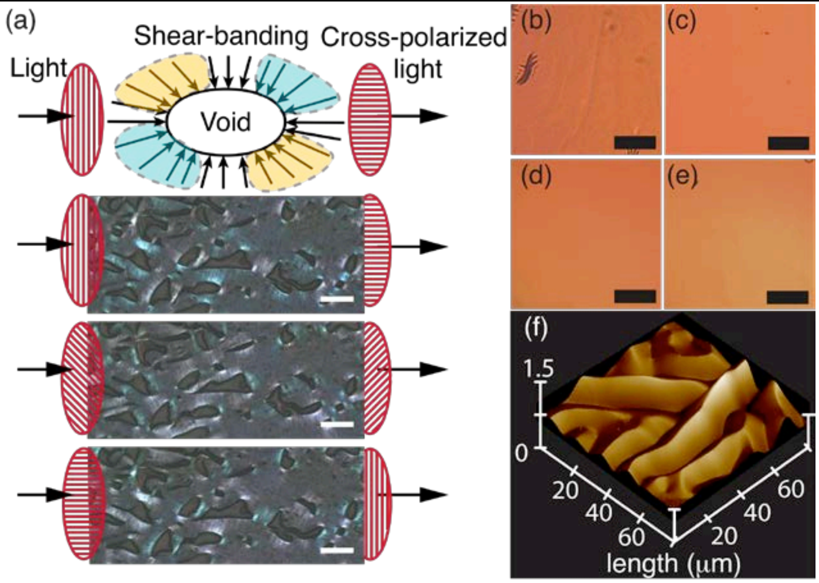

Supplementary Figure 3. (a) Polarized optical micrographs showing radial shear-banding around microvoids in the polymer film exposed by etching off the metal film from the Cu fracture surfaces of polymer-Ta-Cu-MPTMS-silica structures. Optical micrographs from Cu fracture surfaces obtained by fatigue of (b) structures with non-modified Cu-SiO_2 interfaces and (c) polymer-Ta-Cu-MPTMS-silica structures using a harder EPO-TEK 375 polymer instead of the T88 epoxy; and static loading of (d) polymer-Ta-Cu-MPTMS-silica structures, and (e) polymer-Ta-Cu-MPTMS-silica structures followed by 10^5 load cycling at 100 Hz (scale bars are 100 μm). (f) Representative AFM image of Cu fracture surface obtained by fatigue of stacks with Cu-MPTMS-SiO_2 interfaces. All the above data were obtained for p_{H_2O} = 1.3 kPa.

Analyses of mechanical properties and responses

For our Si-polymer-metal-silica-Si beams, the strain rate[7] $\dot{\epsilon} = \nu \frac{3Pl}{Ebh^2}$, where ν is the frequency, *P* the maximum load, *l* the support-to-loading points pin-spacing, *E* the Si elastic modulus, *h* the Si wafer thickness, and *b* the sample width. Inserting *P*=13 N, *l*= 4 mm, *E*= 169 GPa, *b*= 5.6 mm *h*= 630 μm, we find that our observed toughening in $50 \leq \nu \leq 300$ Hz corresponds to $0.02 \leq \dot{\epsilon} \leq 0.1$ s^{-1} at which epoxy T88 constituents exhibit toughening[8–11].

At a metal-polymer interface, the metal wrinkling plastic energy $U_m = \frac{1}{2} E_{\mathrm{m}} h_{\mathrm{m}} \chi_{\mathrm{Wrinkle}} \varepsilon^2$, where the metal film strain[12] $\varepsilon = \frac{\pi^2 A^2}{\lambda^2} + \frac{1}{4}(\frac{3E_{\mathrm{p}}}{E_{\mathrm{m}}})^{2/3}$, E_{m} and E_{p} are metal and polymer bulk moduli, h_{m} the metal thickness, *A* the wrinkle amplitude, λ the wrinkle wavelength, and χ_{Wrinkle} the wrinkle coverage. We get $U_{\mathrm{m}} \sim 0.02 \pm 0.01$ Jm^{-2} for E_{m}= 127 GPa, E_{p}= 7 MPa, h_{m}= 145 nm, *A*= 0.7 μm, λ= 20 μm and χ_{Wrinkle}= 0.6. Tapping-mode AFM was used to measure *A*, and verify λ determined from the optical micrographs (Supplementary Figure 3).

Straining rectangular beams of the polymer epoxy T88 to 0.1% in a Rheometric Scientific DMTA V dynamic mechanical analysis instrument reveal decreases in both storage and loss moduli (*E*' and *E*") with increasing temperature and decreasing frequency, while the tanδ increases (Supplementary Figure 4). Increasing the frequency shifts the loss/storage moduli ratio tanδ curve and the T_g to higher temperatures, by ~5 °C per decade.

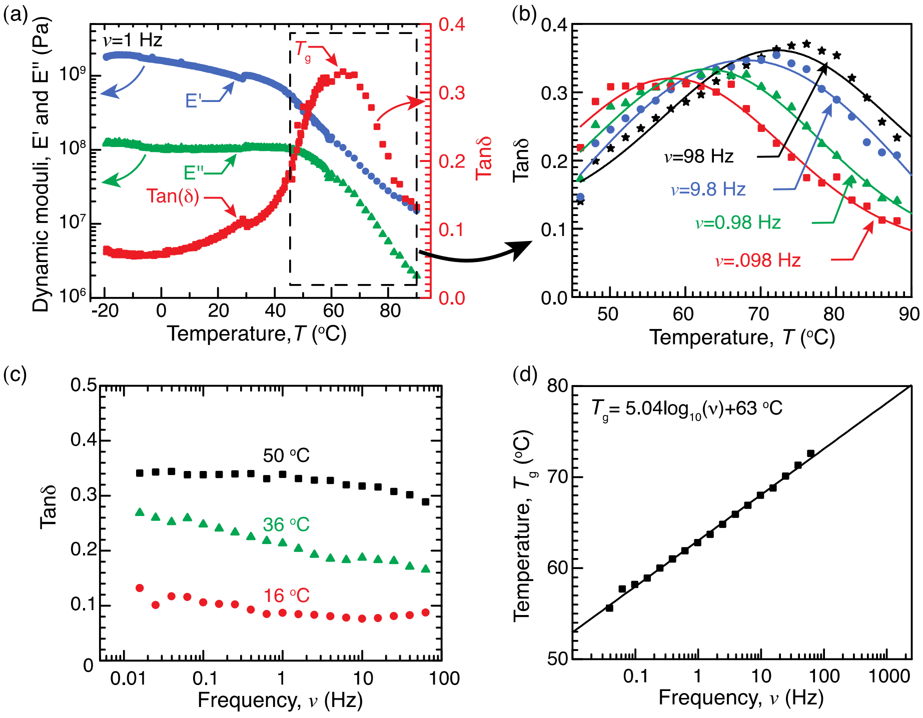

Supplementary Figure 4. (a) Storage and loss moduli (*E*', and *E*", respectively) and loss ratio tanδ (=*E*"/*E*') for the T88 polymer epoxy, measured by dynamic mechanical analysis, plotted as a function of temperature *T* at constant loading frequency of *ν* =1 Hz. The glass transition temperature T_g corresponds to the tanδ maximum. Plots of (b) tanδ - temperature characteristics for different frequencies, (c) tanδ - frequency characteristics for *T*=16 °C, 36 °C, and 50 °C, and (d) T_g – frequency characteristics.

We estimated the contribution of moisture-induced polymer swelling to the observed fracture toughening by carrying out four-point-bend tests on blanket T88 polymer films deposited on silica-capped non-notched silicon wafers for 0.6 kPa ≤ p_{H_2O} ≤ 1.8 kPa (Supplementary Figure 5). Our results reveal a ~0.04 μmN^{-1} compliance decrease attributable to moisture-induced polymer swelling, which is <3% of the ~1.5 μmN^{-1} compliance decrease observed in our fatigue fracture tests. Thus, moisture-induced polymer swelling is not a significant contributor to the observed fatigue fracture toughening.

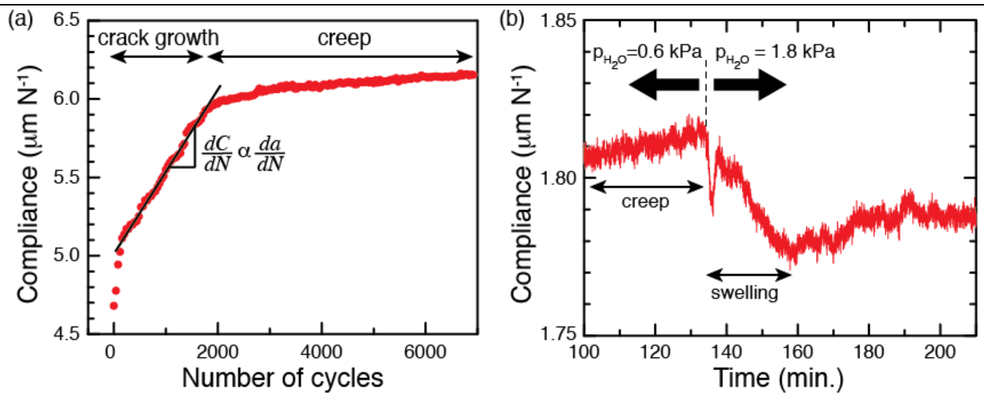

Supplementary Figure 5. Compliance of polymer-Si structures plotted as a function of (a) number of load cycles during fatigue loading, and (b) time, as humidity is tripled. Water-induced polymer stiffening results in a compliance decrease that is <3% of the compliance change observed during our fatigue tests.

Supplementary References

1. Kwan, M., Braccini, M., Jain, A., Lane, M. W. & Ramanath, G. Interplay between bond breaking and plasticity during fracture at a nanomolecularly-modified metal-ceramic interface. *Scr. Mater.* **121,** 42–44 (2016).

2. Gandhi, D. D. *et al.* Annealing-induced interfacial toughening using a molecular nanolayer. *Nature* **447,** 299–302 (2007).

3. Jain, A. *et al.* Atomistic fracture energy partitioning at a metal-ceramic interface using a

nanomolecular monolayer. *Phys. Rev. B* **83,** 35412 (2011).

4. Ma, Q. A four-point bending technique for studying subcritical crack growth in thin films and at interfaces. *J. Mater. Res.* **12,** 840–845 (1997).

5. Ramanath, G. *et al.* Self-assembled subnanolayers as interfacial adhesion enhancers and diffusion barriers for integrated circuits. *Appl. Phys. Lett.* **83,** 383 (2003).

6. Whitesides, G. M., Bowden, N., Brittain, S., Evans, A. G. & Hutchinson, J. W. Spontaneous formation of ordered structures in thin films of metals supported on an elastomeric polymer. *Nature* **393,** 146–149 (1998).

7. Charalambides, P. G., Lund, J., Evans, A. G. & McMeeking, R. M. A Test Specimen for Determining the Fracture Resistance of Bimaterial Interfaces. *J. Appl. Mech.* **56,** 77 (1989).

8. Jiang, W. *et al.* Loading rate dependence of mode II fracture behavior in interleaved carbon fibre/epoxy composite laminates. *Appl. Compos. Mater.* **8,** 361–369 (2001).

9. Todo, M., Takahashi, K., Béguelin, P. & Kausch, H. H. Strain-rate dependence of the tensile fracture behaviour of woven-cloth reinforced polyamide composites. *Compos. Sci. Technol.* **60,** 763–771 (2000).

10. Kusaka, T., Horikawa, N. & Masuda, M. Low-velocity impact fracture behaviour of impact-resistant polymer matrix composite laminates under mixed mode loading. *Le J. Phys. IV* **10,** Pr9-317-Pr9-322 (2000).

11. Vu-Khanh, T. & Fisa, B. Impact fracture of glass-flake reinforced polypropylene. *Polym. Compos.* **7,** 375–382 (1986).

12. Chung, J. Y., Nolte, A. J. & Stafford, C. M. Surface Wrinkling: A Versatile Platform for Measuring Thin-Film Properties. *Adv. Mater.* **23,** 349–368 (2011).
